# Refining the Classroom: The Self-Supervised Professor Model for Improved Segmentation of Locally Advanced Pancreatic Ductal Adenocarcinoma

**DOI:** 10.1007/s10278-025-01555-x

**Published:** 2025-09-23

**Authors:** Jacqueline I. Bereska, Selina Palic, Leonard F. Bereska, Efstratios Gavves, C. Yung Nio, Marnix P. M. Kop, Femke Struik, Freek Daams, Martijn A. van Dam, Tom Dijkhuis, Marc G. Besselink, Henk A. Marquering, Jaap Stoker, Inez M. Verpalen

**Affiliations:** 1 Cancer Center Amsterdam, Amsterdam, the Netherlands; 2 Department of Radiology and Nuclear Medicine, Amsterdam UMC, University of Amsterdam, Amsterdam, the Netherlands; 3 Department of Biomedical Engineering and Physics, Amsterdam UMC, University of Amsterdam, Amsterdam, the Netherlands; 4 Video and Image Sense Lab, University of Amsterdam, Amsterdam, the Netherlands; 5 Department of Surgery, Amsterdam UMC, Vrije Universiteit Amsterdam, Amsterdam, the Netherlands; 6 Department of Surgery, Leiden University Medical Center, Leiden, the Netherlands; 7 Amsterdam Gastroenterology Endocrinology and Metabolism, Amsterdam, the Netherlands

**Keywords:** Pancreatic neoplasms, Carcinoma, Pancreatic ductal, Artificial intelligence, Deep learning

## Abstract

Pancreatic ductal adenocarcinoma (PDAC) is a leading cause of cancer-related deaths, with accurate staging being critical for treatment planning. Automated 3D segmentation models can aid in staging, but segmenting PDAC, especially in cases of locally advanced pancreatic cancer (LAPC), is challenging due to the tumor’s heterogeneous appearance, irregular shapes, and extensive infiltration. This study developed and evaluated a tripartite self-supervised learning architecture for improved 3D segmentation of LAPC, addressing the challenges of heterogeneous appearance, irregular shapes, and extensive infiltration in PDAC. We implemented a tripartite architecture consisting of a teacher model, a professor model, and a student model. The teacher model, trained on manually segmented CT scans, generated initial pseudo-segmentations. The professor model refined these segmentations, which were then used to train the student model. We utilized 1115 CT scans from 903 patients for training. Three expert abdominal radiologists manually segmented 30 CT scans from 27 patients with LAPC, serving as reference standards. We evaluated the performance using DICE, Hausdorff distance (HD95), and mean surface distance (MSD). The teacher, professor, and student models achieved average DICE scores of 0.60, 0.73, and 0.75, respectively, with significant boundary accuracy improvements (teacher HD95/MSD, 25.71/5.96 mm; professor, 9.68/1.96 mm; student, 4.79/1.34 mm). Our findings demonstrate that the professor model significantly enhances segmentation accuracy for LAPC (*p* < 0.01). Both the professor and student models offer substantial improvements over previous work. The introduced tripartite self-supervised learning architecture shows promise for improving automated 3D segmentation of LAPC, potentially aiding in more accurate staging and treatment planning.

**Supplementary Information:**

The online version contains supplementary material available at 10.1007/s10278-025-01555-x.

## Introduction

Pancreatic ductal adenocarcinoma (PDAC), the fourth most common cause of cancer mortality, has a 5-year survival rate of only 5% [1]. Accurate staging of PDAC is crucial for determining treatment options and relies on assessing tumor involvement with critical abdominal vessels, such as the superior mesenteric artery and superior mesenteric vein. Precise automated 3D segmentations of the tumor and adjacent vasculature can assist clinicians in evaluating the tumor’s proximity to vital vessels and aid in surgical planning [2, 3]. However, locally advanced pancreatic ductal adenocarcinoma (LAPC), a more advanced stage of PDAC, presents considerable challenges for segmentation due to extensive tumor infiltration, hazy density around peripancreatic vessels, irregular shapes, and anatomical variability of peripancreatic vasculature among patients [4]. These complexities, coupled with the scarcity of well-annotated data, as manual annotation is exceptionally labor-intensive, time-consuming, and demands substantial expertise in abdominal radiology, underscore an urgent need for innovative solutions capable of accurately segmenting LAPC, even with limited annotated data.

Previous studies leveraging deep learning techniques for the automatic segmentation of PDAC have primarily focused on smaller tumors confined within the pancreas, neglecting LAPC [2, 5–9]. Recent studies have adopted a self-supervised learning framework, where an initial *teacher model* is trained on manually segmented data and subsequently employed to generate pseudo-segmentations—segmentations derived from the model’s predictions rather than human expertise—on a more extensive database, forming the training foundation for a final *student model* [7, 10, 11]. While this approach has demonstrated the potential to boost performance and reduce the need for laborious manual segmentations, the pseudo-segmentations generated by the teacher model for LAPC are often noisy, preventing the student model from learning effectively [10]. The complexity and variability of LAPC cases demand a more robust and adaptive solution that can improve noisy pseudo-segmentations and deliver high-quality segmentations.

To address the limitations of the current self-supervised teacher–student framework, we propose a novel methodology that introduces a third component, the *professor model*, into the framework. The professor model is designed to correct the pseudo-segmentations generated by the teacher model before they serve as training data for the student model. By refining the pseudo-segmentations, the professor model aims to provide the student model with higher-quality training data, ultimately leading to improved segmentation performance for LAPC cases. Accurate segmentation not only assists in staging and surgical planning but also serves as the essential foundation for downstream clinical applications including treatment response prediction, survival analysis, and quantitative assessment of vascular involvement.

## Materials and Methods

The Medical Ethics Review Committee of the 1Amsterdam University Medical Centers (UMC) approved this study protocol and waived the need for informed consent. All patients were managed per institutional practices.

### Datasets

This study retrospectively included four datasets comprising 1115 CTs from 931 patients with resectable, borderline resectable, locally advanced PDAC, and 195 CTs from 195 control patients. Table 1 outlines the characteristics of these datasets. The first dataset, REPDAC, represents the Amsterdam UMC and Leiden University Medical Center subset of the PREOPANC trials conducted by the Dutch Pancreatic Cancer Group [12, 13]. The second dataset, LAPC, includes patients from the Dutch Pancreatic Cancer Group LAPC registry [14]. The third dataset, CONTROL, comprises patients without pancreatic abnormalities who underwent CT scans before transcatheter aortic valve implantation. The fourth dataset, MSKCC, is a publicly available dataset from the Memorial Sloan Kettering Cancer Center [15]. We opted for late arterial phase CT scans (LAP-CTs) from the REPDAC, LAPC, and CONTROL datasets due to enhanced tumor and pancreas visibility at this phase, when available. However, for the MSKCC dataset, we utilized portal-venous phase scans (PVP-CT), as these were the only ones available, and two phases provide complementary information in PDAC segmentation [9]. All patients in the REPDAC, LAPC, and CONTROL datasets provided general informed consent.

**Table 1:** Dataset characteristics for the REPDAC, LAPC, CONTROL, and MSKCC datasets

Characteristic	REPDAC	LAPC	CONTROL	MSKCC
Scans	344	126	195	420
Patients	170	118	195	420
Timeframe	2013–2020	2019–2021	2013–2017	N/A
Contrast	Late arterial	Late arterial	Late arterial	Portal venous
Center	Amsterdam UMC, Leiden University Medical Center	Amsterdam UMC	Amsterdam UMC	MSKCC
Abnormality	Resectable and borderline resectable PDAC	LAPC	None	PDAC, intraductal mucinous neoplasms, pancreatic neuroendocrine tumors

*UMC* University Medical Centers, *MSKCC* Memorial Sloan Kettering Cancer Center, *PDAC* pancreatic ductal adenocarcinoma, *LAPC* locally advanced pancreatic ductal adenocarcinoma

### Data Preparation

One of three radiologists (C.Y.N, 27 years’ experience; F.S., 3 years’ experience; and M.K., 6 years’ experience) manually segmented the PDAC tumors in 256 LAP-CTs of 120 patients with (borderline) resectable PDAC (REPDAC dataset) and 66 LAP-CTs of 66 patients with LAPC (LAPC dataset) using a 3D slicer version 4.11.20210226 [16]. This manual segmentation was performed on the original unannotated CT scans to ensure precise delineation of tumor boundaries. In addition, to provide context and improve accuracy by enabling the model to distinguish PDAC from adjacent tissues more effectively, we independently automatically segmented surrounding anatomical structures, the pancreas, duodenum, spleen, kidneys, adrenal glands, liver, and gallbladder, using TotalSegmentator version 1.5.6 [17]. Figure S1 of the Supplement provides an example of a segmented LAP-CT.

### Model Implementation

**Fig. 1:**
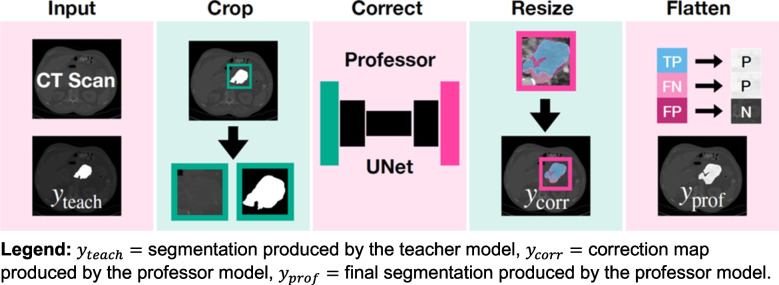
Architectural overview of the proposed professor model for PDAC segmentation refinement. The diagram illustrates the four sequential steps of the segmentation process: (**A**) initial segmentation by the teacher model (y_teach), (**B**) generation of the correction map (y_corr), (**C**) application of the correction matrix, and (**D**) production of the final refined segmentation (y_prof). This framework demonstrates how the professor model systematically improves upon the teacher model’s initial predictions through targeted correction mechanisms.

We propose a professor model for automatically correcting pseudo-segmentations resulting from the teacher model as an addition to the conventional teacher–student framework in deep learning for medical image segmentation. The teacher–professor–student model extends the traditional teacher–student framework by introducing an intermediate refinement step. The professor model acts as a correction mechanism, improving the quality of pseudo-segmentations before they are used to train the student model, thus mitigating the propagation of errors in complex cases like LAPC. An overview of the proposed professor model is provided in Fig. 1.

#### Preprocessing

To increase computational efficiency and reduce irrelevant contextual information, we first cropped the ground-truth and teacher-generated PDAC pseudo-segmentations and CT scans to a bounding box of the union of ground-truth and teacher-generated pseudo-segmentations.

#### Architecture

We used a 3D UNet model architecture for the professor model with an evenly weighted combination of DICE loss and cross-entropy loss and a fivefold cross-validation approach for training [18]. We employed a 3D U-Net architecture due to its ability to effectively capture volumetric spatial context, which is crucial for accurately segmenting the complex and infiltrative nature of pancreatic tumors [18]. We normalized the CT images according to their initial CT normalization applied during the teacher model’s training. The input for the professor model comprised the CT scans and the teacher-generated segmentations. The professor model’s training signals are derived from correction matrices denoted as *y*_corr_, formulated using both the ground-truth *y*_*true*_ and teacher-generated segmentations denoted as *y*_teach_. Specifically, we defined the professor model’s ground truth*y*_*corr*_ as follows:

$${y}_{corr}=\left\{\begin{array}{c} FN, if\ {y}_{teach}\ne {y}_{true}\ and\ {y}_{teach}=0\\ {} FP, if\ {y}_{teach}\ne {y}_{true}\ and\ {y}_{teach}=1\\ {} FN, if\ {y}_{teach}={y}_{true}\ and\ {y}_{teach}=1\\ {}{y}_{teach}, otherwise\end{array}\right.$$

where *y*_teach_, *y*_true_ = 1 for pixels belonging to the PDAC tumor, and *y*_teach_, *y*_true_ = 0 for pixels belonging to the background according to the teacher model and ground truth segmentation, respectively. Here, FN represents false negatives, pixels incorrectly labeled as background but should have been identified as tumor, while FP denotes false positives, pixels erroneously marked as tumor that should have been classified as background. Conversely, TP identifies true positives, pixels accurately identified as tumor. These three categories are distinct, but the correction matrix allows for overlapping values for FN, FP, and TP, enabling various combinations and interpretations. This flexibility facilitates custom adjustments to prioritize specific error types.

**Fig. 2:**
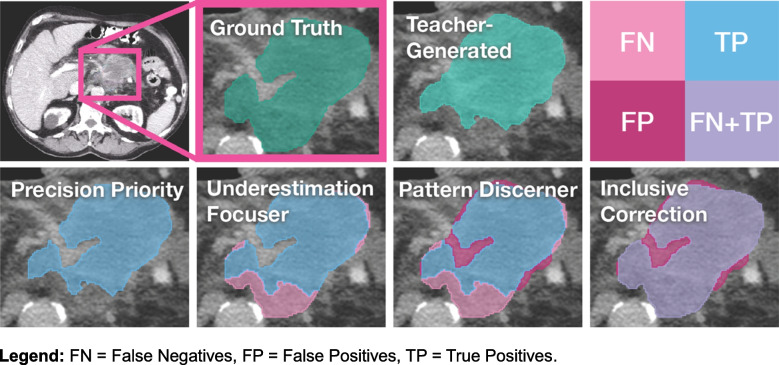
Comparative visualization of correction matrices for LAPC segmentation refinement in arterial phase CT imaging. The figure presents ground-truth segmentations alongside teacher-generated predictions and demonstrates the four distinct correction matrix approaches: Precision Priority (emphasizing true positives), Pattern Discerner (weighted handling of FN = 1, FP = 2, TP = 3), Underestimation Focuser (prioritizing FN = 1, TP = 2), and Inclusive Correction (balanced approach with FN = 2, FP = 1, TP = 2). False negatives (FN), false positives (FP), and true positives (TP) are color-coded to illustrate the differing emphasis of each correction strategy. FN, false negatives; FP, false positives; TP, true positives

We developed and tested four distinct correction matrices: Precision Priority, Pattern Discerner, Underestimation Focuser, and Inclusive Correction, each designed to address different error patterns. Precision Priority focuses on correctly identified tumor pixels (TP = 1), disregarding both types of misclassifications (FN = 0, FP = 0) under the assumption that errors lack systematic patterns for learning enhancement. Pattern Discerner addresses all prediction outcomes (FN = 1, FP = 2, TP = 3), assuming that the teacher model’s overestimations and underestimations exhibit identifiable, correctable patterns. Underestimation Focuser concentrates on correctly identified tumors and underestimations (FN = 1, TP = 2), intentionally omitting overestimations (FP = 0), based on the belief that underestimating tumor presence constitutes the most instructive error category for model refinement. Inclusive Correction focuses both on accurate tumor predictions and areas of overestimation (FN = 2, FP = 1, TP = 2), hypothesizing that the teacher model’s primary errors lie in overestimating tumor regions. A visual example of each correction matrix is provided in Fig. 2.

#### Resizing

We restored the correction matrix to the dimensions of the original segmentation.

#### Correction Matrix Transformation

We applied the predicted correction matrix to correct the teacher-generated segmentations as follows:

$${y}_{prof}=\left\{\begin{array}{c}1, if\ FN\\ {}0, if\ FP\\ {}1, if\ FP\\ {}{y}_{teach}, otherwise.\end{array}\right.$$

### Model Training

**Fig. 3:**
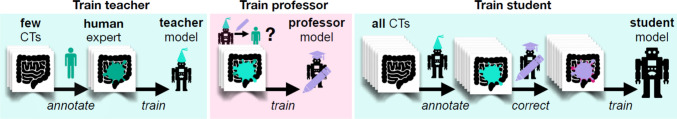
Comprehensive workflow diagram of the enhanced self-supervised learning framework for PDAC segmentation. The schematic details the three-phase training process: (1) teacher model training on manually segmented data, (2) professor model training for segmentation refinement, and (3) student model training using the refined segmentations. This diagram illustrates the novel integration of semi-supervised and self-supervised learning approaches, highlighting the unique role of the professor model in improving segmentation quality

The training was structured into three phases [1] training the teacher model on a small set of manually segmented data to segment PDAC, [2] training the professor model on a small set of manually segmented data to correct the teacher model’s segmentations, and [3] training the student model on the entire training set segmented by the teacher model and corrected by the professor model to segment PDAC. These steps are described in Fig. 3. This progressive approach allows each model to build upon the knowledge of its predecessors while maintaining specialization in their respective tasks. While this approach incorporates elements of self-supervised learning, particularly in the teacher–student framework, it also shares characteristics with semi-supervised methods. The initial training of the teacher model on manually labeled data, followed by the use of unlabeled data for the student model, aligns more closely with semi-supervised paradigms. However, the introduction of the professor model for refining pseudo-labels introduces a self-supervised component. This hybrid approach leverages the strengths of both methodologies: the ability to learn from limited labeled data (semi-supervised) and the capacity to improve representations without additional human annotation (self-supervised).

#### The Teacher Model

Training consisted of 517 CTs described in Table 2 with manual segmentations of the PDAC tumor if present and automatic segmentations of surrounding anatomical structures obtained from TotalSegmentator [17]. The teacher model comprised a simple nnUNet architecture involving a two-stage 3D UNet cascade [19]. The initial UNet in this setup was trained on downsampled images to create low-resolution segmentations. These low-resolution outputs were then used as additional inputs for training the full-resolution UNet. The low-resolution stage processed inputs at approximately 2.85 × 1.45 × 1.45 mm spacing with patch sizes of 96 × 160 × 160 voxels, while the full-resolution stage operated at 2.0 × 0.71 × 0.71 mm spacing with patch sizes of 64 × 192 × 160 voxels. Both stages utilized a base feature count of 32 channels in the initial layer, increasing to a maximum of 320 channels in the deepest layers. The network architecture consisted of six encoder stages and five decoder stages, with two convolutional layers per stage (3 × 3 × 3 kernels). The downsampling path employed pooling operations with varying strides across different axes, optimized for the anisotropic resolution of CT scans.

**Table 2:** Training sets for the Teacher, Professor, and Student models

Dataset	Teacher model	Professor model	Student model
REPDAC			
Number of scans	256 LAP-CTs	40 LAP-CTs	344 LAP-CTs
Number of patients	120	40	170
LAPC			
Number of scans	66 LAP-CTs	66 LAP-CTs	126 LAP-CTs
Number of patients	58	58	118
CONTROL			
Number of scans	195 LAP-CTs	--	195 LAP-CTs
Number of patients	195	--	195
MSKCC			
Number of scans	--	--	420 PVP-CTs
Number of patients	--	--	420
Total			
Number of scans	517 LAP-CTs	106 LAP-CTs	1085 CTs
Number of patients	373	98	903

*LAP-CT* late arterial phase CT scan, *PVP-CT* portal venous phase CT scan

#### The Professor Model

Training consisted of 106 CTs described in Table 2 with PDAC segmentations generated by the teacher model during the teacher model’s cross-validation across five training folds. We excluded the CONTROL scans as these do not contain a tumor and reduced the number of REPDAC scans to focus on LAPC specifically. The professor model used a standard UNet architecture, operating at a spacing of 2.0 × 0.70 × 0.70 mm with patch sizes of 24 × 64 × 80 voxels. This model processed both the original CT data and the teacher’s predictions as dual-channel input (CT intensities and segmentation masks). The network consisted of five encoder stages and four decoder stages with two convolutional layers per stage, using 3 × 3 × 3 kernels. The professor model employed asymmetric pooling strategies with two pooling operations along the z-axis and four pooling operations along the x and y axes. The specific pooling kernel sizes were [1, 1, 1], [1, 2, 2], [2, 2, 2], [2, 2, 2], and [1, 2, 2] for the five stages, preserving z-axis resolution in the first, second, and final pooling stages. We selected the correction matrix of the best-performing professor model for segmenting LAPC during the fivefold cross-validation.

#### The Student Model

Training consisted of 1085 CTs from 903 patients described in Table 2. First, we used the teacher model to segment 568 CTs of this dataset for which no manual segmentations were available. Second, we used the professor model to correct these PDAC segmentations. Finally, we trained the student model with the resulting teacher-segmented and professor-corrected CTs and the manually segmented 517 CTs used to train the teacher model. We employed the same 3D UNet cascade network architecture for the student and teacher model, with slight differences in configuration parameters. The low-resolution stage operated at 2.53 × 1.30 × 1.30 mm spacing with patch sizes of 64 × 192 × 192 voxels, while the full-resolution stage processed inputs at 2.0 × 0.76 × 0.76 mm with patch sizes of 48 × 192 × 192 voxels. The student model maintained the same network design principles, with six encoder stages, five decoder stages, 32 base features increasing to 320 maximum features, and identical convolution kernel sizes.

### Training Hyperparameters

All models were trained using the SGD optimizer (momentum = 0.99, nesterov = true, weight_decay = 3e−5) with an initial learning rate of 0.01 and polynomial decay. We employed a fivefold cross-validation approach using a combined Dice and cross-entropy loss function with deep supervision. Training continued for 1000 epochs with 250 iterations per epoch, and validation was performed every 50 iterations. The data augmentation pipeline included spatial transformations (rotations ± 30°, scaling 0.7–1.4), Gaussian noise, Gaussian blur, brightness and contrast adjustments, and mirroring along all axes. We used a batch size of 2 for the teacher and student models and 3 for the professor model, constrained by GPU memory.

### Performance Evaluation

We used multiple metrics to comprehensively evaluate segmentation performance. The Dice Similarity Coefficient (DSC) measured overall segmentation accuracy through volumetric overlap. To assess boundary accuracy, we calculated Hausdorff Distance 95 (HD95), which quantifies the 95 th percentile of the maximum surface distance between segmentations, and Mean Surface Distance (MSD), which measures the average minimum distance between segmentation surfaces. Additionally, we computed sensitivity (proportion of actual tumor voxels correctly identified) and specificity (proportion of non-tumor voxels correctly identified) to evaluate the models’ clinical utility in detecting tumors and sparing healthy tissue.

We evaluated all three models on a test dataset containing 30 randomly selected CTs from 27 patients from the LAPC dataset that were not used to train the teacher, professor, or student segmentation model. To address patient-level correlation in our dataset, we ensured all scans from the same patient were assigned to a single partition (training or testing). We applied a Wilcoxon signed-rank test to assess the statistical significance of performance differences between teacher-generated and professor-corrected segmentations across all metrics. A *p*-value of 0.05 was considered statistically significant.

## Results

### Patient Characteristics

The test set contained 30 CTs from 27 patients with LAPC. The test set comprised nine females (33%) and 18 males (66%) with a median tumor diameter of 4.0 cm (interquartile range of 1.2 cm) and a maximum diameter of 8.6 cm.

### Segmenting Locally Advanced Pancreatic Ductal Adenocarcinoma

During training, the professor model trained with the correction matrix Underestimation Focuser achieved the highest fivefold cross-validation DSC. As a result, we selected the professor model trained with the Underestimation Focuser correction matrix as our final professor model. Table S1 of the Supplement outlines the results of the fivefold cross-validation training of the professor model.

**Fig. 4:**
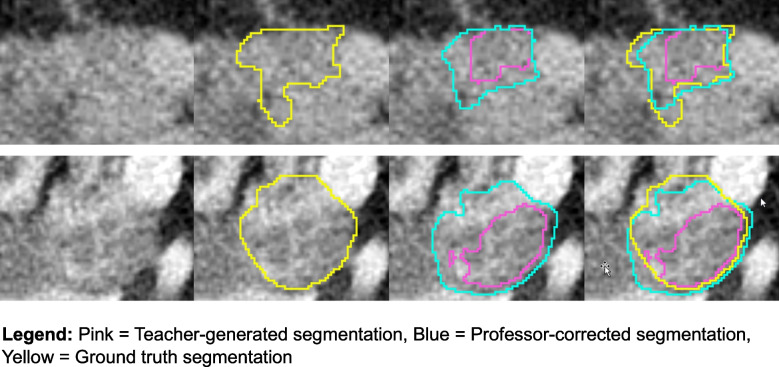
Multi-model segmentation comparison of LAPC in an arterial phase CT imaging. The figure demonstrates the progressive improvement in segmentation accuracy across three models: teacher-generated segmentation (pink), professor-corrected segmentation (blue), and ground truth segmentation (yellow). This visual comparison illustrates the enhanced accuracy achieved through the professor model’s refinement process, particularly in areas of complex tumor margins and vessel involvement. The color coding enables direct visual assessment of segmentation improvements and remaining discrepancies

On the test set, the teacher and professor model achieved an average DSC of 0.60 (standard deviation) (dev, 0.16) and 0.73 (dev, 0.13), respectively. The Wilcoxon signed-rank test results revealed a significant difference between teacher-generated and professor-corrected segmentations in terms of DSC (*p* < 0.05). The professor model yielded a median increase in terms of DSC of 0.14 (max, 0.28; min, −0.5; median absolute deviation, 0.05). The student model trained with the professor-corrected segmentations achieved a significantly higher DSC of 0.74 (dev, 0.13) compared to the professor model (*p* < 0.05). Boundary accuracy metrics demonstrated substantial improvements, with the professor model achieving a mean Hausdorff Distance 95 (HD95) of 9.68 ± 9.13 mm compared to the teacher model’s 25.71 ± 13.89 mm, representing a 62% reduction in boundary error. Similarly, mean surface distance (MSD) improved from 5.96 ± 3.14 mm with the teacher model to 1.96 ± 1.09 mm with the professor model. The professor model also maintained high sensitivity (0.9563 ± 0.0597) and specificity (0.9995 ± 0.0010), compared to the teacher model’s sensitivity of 0.9437 ± 0.0665 and specificity of 0.9993 ± 0.0008. The student model trained with the professor-corrected segmentations achieved a significantly higher DSC of 0.74 (dev, 0.13) compared to the professor model (*p* < 0.05), and further improved boundary accuracy with HD95 of 4.79 ± 1.32 mm and MSD of 1.34 ± 0.41 mm, while maintaining high sensitivity (0.9051 ± 0.1068) and specificity (0.9996 ± 0.0007). Figure 4 provides an example of segmentations generated by each model.

## Discussion

This study proposed an iterative learning schema for segmenting locally advanced pancreatic ductal adenocarcinoma. The approach integrates a tripartite model architecture: a teacher model trained on manually segmented CT scans to generate preliminary pseudo-segmentations, a professor model that refines these segmentations, and a student model that uses refined segmentations for training. The qualitative analysis demonstrated that the segmentations refined by the professor model were superior to those generated by the teacher model, displaying significantly higher DSC. Additionally, the final student model surpassed both the teacher and professor models in terms of segmentation accuracy. The results indicate that the proposed iterative learning schema effectively improves segmentation accuracy for LAPC while minimizing the need for manual segmentations.

Existing PDAC tumor segmentation models have achieved DSC spanning from 0.57 to 0.71. Mahmoudi et al.’s [2] integration of deep convolutional neural networks with texture descriptors resulted in a DSC of 0.6, whereas Zhu et al. [5] achieved a DSC of 0.57 through a multiscale, coarse-to-fine model. Furthermore, Zhang et al.’s [7] exploration into a teacher–student self-supervised learning methodology attained a DSC of 0.71. Notably, these studies, while promising, have not reported DSC specific to LAPC, with Zhu et al. [5] documenting a mean tumor size of 2.5 cm and Zhou et al. [6] excluding cases exceeding 4 cm. Despite our evaluation of LAPC cases, acknowledged as challenging for both radiologists and segmentation models, we report a substantial improvement in terms of DSC compared to previous efforts. Our evaluation further demonstrated that boundary accuracy progressively improved from teacher to professor to student model, with substantial reductions in border delineation errors while maintaining excellent sensitivity and specificity, confirming that our tripartite architecture enhances both volumetric overlap and contour precision for clinical applications.

One limitation of our approach is the need for increased computational resources during training due to incorporating an additional model. Furthermore, the correction matrix we utilized, the Underestimation Focuser, might not be universally applicable across different types of segmentation challenges, necessitating multiple professor models to accommodate diverse scenarios and further compounding computational demands. An additional concern arises from the reliance on imperfect ground truth segmentations for training the teacher and professor model. Given the complexity of accurately delineating PDAC—a task that becomes even more challenging with LAPC—the ground truth segmentations, not verified by pathology, may inadvertently include fibrotic tissue alongside cancerous regions. Additionally, our use of single-radiologist segmentations, while maximizing data quantity, may introduce further inaccuracies. Although the Underestimation Focuser emerged as the most effective correction matrix in our experiments, there is a possibility that the ostensibly enhanced tissue delineation by the professor model includes fibrosis, a hypothesis that necessitates pathological refutation. Future work should evaluate the student model on external center data to determine the robustness of the results. Framework generalizability should be assessed using alternative UNet variants such as ResUNet, DenseUNet, or UNet++ as professor models. Additionally, exploring the direct incorporation of the professor loss into the teacher UNet model warrants further investigation.

In conclusion, this study makes three key contributions to LAPC segmentation: (1) introduction of a novel tripartite self-supervised learning architecture that significantly improves segmentation accuracy with a DSC of 0.75, surpassing previous approaches that achieved DSC values of 0.57–0.71; (2) successful targeting of challenging LAPC cases with complex morphology and extensive infiltration that have been largely overlooked in prior research; and (3) development of an effective correction matrix methodology, particularly the Underestimation Focuser approach, which systematically refines imperfect segmentations by prioritizing missed tumor regions. These segmentation advancements have immediate clinical relevance as accurate LAPC segmentation serves as the essential foundation for numerous AI-driven applications including surgical planning through precise vascular involvement assessment, development of predictive models for treatment response and survival outcomes, and quantitative monitoring of tumor changes during therapy, ultimately contributing to better patient outcomes.

## Supplementary Information

ESM 1 (DOCX 334 kb)

## Data Availability

The authors confirm that the data supporting the findings of this study are available within the article and its supplementary material. The documented code, fully trained models, and test set will be made available on GitHub (https://github.com/JackieBereska/PDACSegmentation) and Zenodo (https://zenodo.org/records/14782552) upon publication.

## Abbreviations

PDAC: Pancreatic ductal adenocarcinoma
LAPC: Locally advanced pancreatic ductal adenocarcinoma
LAP-CT: Late arterial phase CT scan
PVP-CT: Portal venous phase CT scan
TP: True positive
FP: False positive
TN: True negative
FN: False negative
DSC: Dice similarity coefficient
