# Supplementary material for: Refining the Classroom: The Self-Supervised Professor Model for Improved Segmentation of Locally Advanced Pancreatic Ductal Adenocarcinoma
Source: J Imaging Inform Med. 2025 Sep 23;39(3):2330–9. doi: 10.1007/s10278-025-01555-x (PMC13230426; doi:10.1007/s10278-025-01555-x)
Supplement: Supplementary file 1 — (DOCX 334 kb) [file 10278_2025_1555_MOESM1_ESM.docx]

**FIGURES**

**Figure 1:** Example of a late arterial phase CT scan (left) with manual segmentations of the pancreatic ductal adenocarcinoma tumor and automatic segmentations of surrounding abdominal organs.


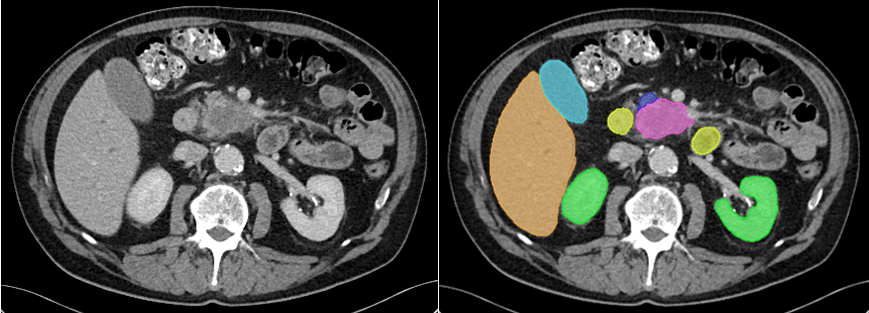


**Legend:** Pink: Pancreatic Ductal Adenocarcinoma Tumor, Orange = Liver, Green = Kidneys, Yellow = Duodenum, Light Blue = Gallbladder, Dark Blue = Pancreas.

**Table 1:** Five-fold cross-validation results for each of the four correction matrices.

| **Performance Metric** | **Precision Priority** | **Inclusive Correction** | **Pattern Discerner** | **Underestimation  Focuser** | |
| --- | --- | --- | --- | --- | --- |
| Dice Similarity Coefficient | 0.67 | 0.69 | 0.69 | | **0.70** |
